# Resampling Nucleotide Sequences with Closest-Neighbor Trimming and Its Comparison to Other Methods

**DOI:** 10.1371/journal.pone.0057684

**Published:** 2013-02-27

**Authors:** Kouki Yonezawa, Manabu Igarashi, Keisuke Ueno, Ayato Takada, Kimihito Ito

**Affiliations:** 1 Department of Computer Bioscience, Nagahama Institute of Bio-science and Technology, Nagahama, Shiga-pref, Japan; 2 Division of Bioinformatics, Hokkaido University Research Center for Zoonosis Control, Kita-ku, Sapporo, Japan; 3 Division of Global Epidemiology, Hokkaido University Research Center for Zoonosis Control, Kita-ku, Sapporo, Japan; George Mason University, United States of America

## Abstract

A large number of nucleotide sequences of various pathogens are available in public databases. The growth of the datasets has resulted in an enormous increase in computational costs. Moreover, due to differences in surveillance activities, the number of sequences found in databases varies from one country to another and from year to year. Therefore, it is important to study resampling methods to reduce the sampling bias. A novel algorithm–called the closest-neighbor trimming method–that resamples a given number of sequences from a large nucleotide sequence dataset was proposed. The performance of the proposed algorithm was compared with other algorithms by using the nucleotide sequences of human H3N2 influenza viruses. We compared the closest-neighbor trimming method with the naive hierarchical clustering algorithm and 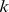-medoids clustering algorithm. Genetic information accumulated in public databases contains sampling bias. The closest-neighbor trimming method can thin out densely sampled sequences from a given dataset. Since nucleotide sequences are among the most widely used materials for life sciences, we anticipate that our algorithm to various datasets will result in reducing sampling bias.

## Introduction

When investigating the transmission of an infectious disease, researchers utilize the similarity among nucleotide sequences of its causative agent. Remarkable efforts have been made at both the national and international levels to collect genetic information on important pathogens. As a result, a large number of pathogen-related sequences have been accumulated in public databases. There exist more than 170000 nucleotide sequences of influenza viruses in the NCBI Influenza Virus Resources [1] and more than 410000 sequences of human immunodeficiency viruses in the HIV sequence database [2].

The rapid growth in the number of nucleotide sequences poses two critical problems. One is an enormous increase in computational costs. Sequence data analyses–including the multiple sequence alignment, phylogenetic analysis, and similarity searches of nucleotide sequences–involve time-consuming computations. Multiple sequence alignment is an NP-complete problem [3]. Phylogenetic analysis using the neighbor-joining method takes 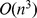 time, where 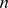 denotes the number of sequences [4]. The similarity searches using BLAST take 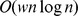 time, where 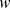 and 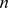 denote the length of the subsequence of queries and the number of sequences, respectively [5].

The other problem is sampling bias in public databases, which occurs when sequences are not sampled randomly. One factor is the difference in surveillance activities among countries. Developed countries having high surveillance activities submit more sequences than other countries. Another factor is the advance in sequencing technologies in the last two decades. The databases tends to contain more sequences from recent strains than from old strains. Therefore it is important to study resampling methods to reduce sampling bias.

There are several methods that might be used for resampling tasks. Zaslavsky et al. proposed a resampling method that was used to display large phylogenetic trees in a limited screen area [6]. Some clustering algorithms, including naive hierarchical clustering (cf. [7]) and 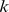-medoids clustering [8], select certain data points as representatives of clusters. These clustering algorithms can be used for resampling large datasets. One simple idea to reduce sampling bias is to remove more sequences from densely sampled ones than from sparsely sampled ones.

In this paper we propose a novel algorithm–called the closest-neighbor trimming method–that resamples a given number of sequences from a large nucleotide sequence dataset. The method first constructs a phylogenetic tree with the whole sequence dataset. It finds the pair of neighbors having the shortest distance among all pairs of neighbors, and trims one leaf away. By repeating this procedure, the algorithm thins out densely sampled sequences in the dataset. We compare the performance of the closest-neighbor trimming method with those of other methods with respect to the average maximum similarities of discarded sequences to the resampled sequences, nucleotide diversities of the resampled sequences, and standard deviations for the number of resampled sequences in a year.

## Materials and Methods

### The Resampling Problem

Before describing the algorithms, we define the resampling problem. Given a set of 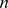 sequences 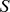, resampling is a task to select a subset of 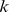 sequences 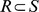. We assume that all the sequences in 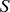 are of the same length or already aligned. 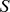 might not be randomly sampled from the population. The characteristics of sequences in the population are often unknown except for some features. The goal of resampling is to find 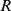, which reflects original characteristics of sequences in the population.

### The Closest-neighbor Trimming (CNT) Method

**Figure 1:**
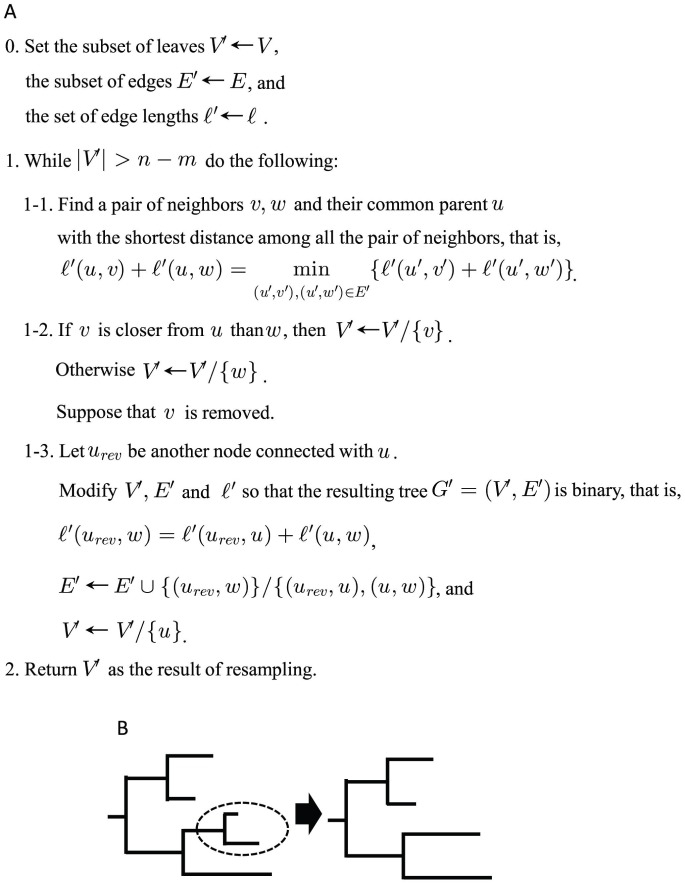
Description of the CNT algorithm. (A) the pseudocode and (B) a schematic image.

We propose a resampling algorithm–called the closest-neighbor trimming (CNT) method–that removes densely sampled sequences. First, CNT constructs a phylogenetic tree from all the sequences in the dataset. CNT does not assume a particular tree construction method. If the phylogenetic tree is not binary, CNT arbitrarily arranges the tree so that it is binary. We denote a binary phylogenetic tree by 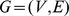, where 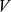 and 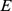 represent a set of nodes and a set of edges, respectively. Given 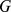, CNT repeats the following procedures until the number of remaining sequences reaches 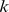. First CNT finds the pair of neighbors with the shortest distance among all pairs of neighbors. Then it removes one of the neighbors. After that it removes the parent and connects the upper branches of the remaining leaf so that the resulting tree 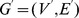 is binary. Whether the CNT removes one of the neighbors further from their parent or one closer to it can be specified. In this paper, we call it the CNT-closer algorithm when it trims the neighbor further from the parent, that is, it leaves the closer one. We call it the CNT-further algorithm when it leaves the further neighbor. In the case that it randomly chooses one of the neighbors to be trimmed, it is called the CNT-random algorithm. However, for simplicity we treat only the CNT-further algorithm (CNT for short) in the main paper and all types of CNT algorithms are dealt with in Supporting Information S1. The pseudocode and a schematic image of the CNT-further method are shown in Figure 1.

### Comparison of the CNT Method with Other Methods

#### The method proposed by Zaslavsky et al. (ZAS05)

We denote the algorithm proposed by Zaslavsky et al. [6] as ZAS05. Given a phylogenetic tree 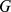, ZAS05 first selects two sequences. One is the closest to the root and the other is the furthest from the root. After that, it selects a sequence at each step until the number of selected sequences reaches 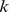 as follows: Suppose 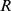 is the set of already selected sequences. For sequence 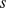, the distance between 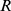 and 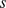 is defined as the minimum distance between 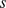 and the one belonging to 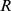. Their algorithm finds the sequence that has the maximum distance to 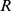.

#### The naive hierarchical clustering (NHC) algorithm

The naive hierarchical clustering (NHC) or UPGMA (cf. [7]) selects some data points as representatives of clusters. By removing the data points other than selected representatives, this algorithm can be used for resampling. Giving an 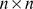 dissimilarity matrix 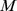, NHC finds the pair having the shortest distance among all the pairs of sequences. Then NHC discards the one having longer distance to all the other sequences. NHC repeats this procedure until the number of remaining sequences reaches 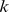. When there is more than one pair with the shortest distance, NHC selects the pair that contains the sequence appearing earliest in the dataset among all the pairs with the shortest distance.

#### The 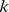-medoids clustering (kMC) algorithm

We also apply the 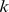-medoids clustering (kMC) method [8] for resampling sequences. Given an 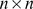 distance matrix 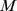, first kMC randomly selects 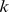 sequences as medoids. Then kMC repeats the following procedures. It assigns each sequence to the closest medoid. For each cluster it updates the medoid so that the total distance from the medoid to other members becomes the smallest. kMC repeats these procedures until no medoids change or the number of repetitions reaches a given threshold (1000 times in this paper).

#### The dataset and the construction of phylogenetic trees

Nucleotide sequences of the hemagglutinin (HA) gene of human H3N2 influenza viruses were downloaded from the NCBI Influenza Virus Resource [1]. The sequences of the HA1 domain were aligned using the MAFFT program [9]. The original dataset included sequences with the ambiguous nucleotide N, making it impossible to calculate the distance matrix of the dataset. Thus the sequences having ambiguous symbols N were removed. After that, we obtained 4655 sequences of 984 nucleotides. The dataset is as the same as that used in [10].

CNT and ZAS05 do not assume a particular method to construct phylogenetic trees. In our analysis the neighbor-joining method [4] was used. PHYLIP [11] was employed for constructing the phylogenetic trees. Here, the Jukes-Cantor model [12] was applied for constructing distance matrices. The resulting phylogenetic tree is shown in Figure 9A. Like this one, phylogenetic trees constructed with nucleotide sequences of influenza A viruses tend to have a very high fraction of sequences having other very similar ones and a characteristically unbalanced distribution of ancestral nodes.

### Evaluation of the Performances of the Resampling Algorithms

#### Average maximum similarities of discarded sequences to the resampled sequences

We need to evaluate the performances of resampling algorithms with respect to preservation the nature of the original dataset and reduction of sampling bias. Regardless how the trimmed sequence data are used, they should cover the original dataset. For this task, we introduced two measurements. The first measure was the average identity from the 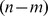 discarded sequences to the 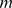 resampled sequences. We denote the number of different nucleotides between sequences 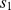 and 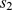 by 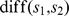. The identity between two sequences 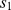 and 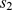, 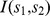, is defined as the ratio of the same nucleotides in the two sequences, that is, 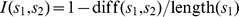. We define the identity from the discarded sequences 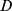 to the resampled sequences 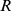 as follows:
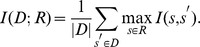



#### Nucleotide diversities of resampled sequences

We introduced nucleotide diversity proposed by Nei and Li [13] for verifying whether the resampled sequences had enough variety. Resampled sequences with low nucleotide diversity may lead to different results from the original sequences. Let 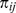 be the number of nucleotide differences per nucleotide site between the 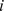
th and 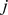
th sequences and 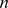 be the total number of nucleotide sequences. Then the nucleotide diversity 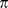 is defined as follows:
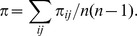



#### Standard deviations for the number of resampled sequences in a year

We utilize the distribution of years when the viruses in the dataset were isolated. In the ideal dataset the number of nucleotide sequences of an organism should be proportional to the number of individuals. In this paper we use a simplified assumption that the dataset should contain equal numbers of sequences. Although the numbers of patients and infection isolates vary extremely from year to year [14], the fluctuation in the number of sequences registered to the databases each year does not seem to be relative to the fluctuation of the number of patients or infection isolates. Thus we use the standard deviation of the number of sequences to evaluate the resampling algorithms with respect to reduction of sampling bias.

## Results

### Distribution of the Sequence Dataset

**Figure 2:**
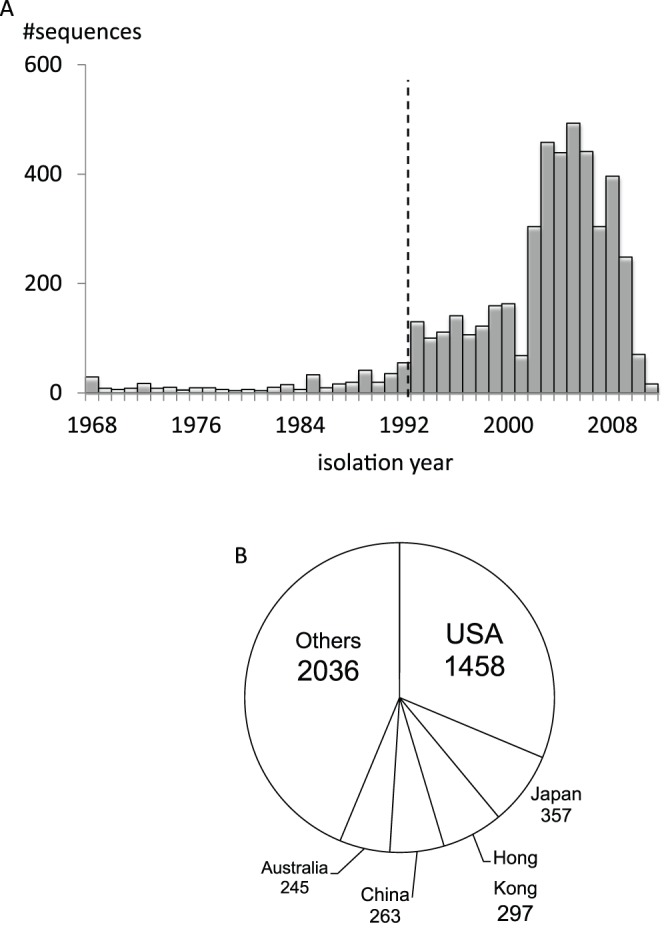
Statistics of the sequences of HA of human H3N2 influenza virus. (A) distribution of isolation years and (B) distribution of isolation countries. More than 92% were sequences isolated after 1992 and more than 30% were sequences of influenza viruses isolated from the USA. Moreover, there is a large gap on the number of nucleotide sequences of HA of human H3N2 influenza virus isolated before 1991 and after 1992, when the PCR technique had been in widespread use.

The dataset contained nucleotide sequences of human H3N2 influenza viruses isolated during the period from 1968 to 2011. Sequences from 1968 to 1991 accounted for about 7% of the dataset and about 93% were sequences from 1992 to 2011 (Figure 2A). This skewed distribution could be attributed to sampling bias due to the rapid development of sequencing technology around 1992 [15]. Additionally, more than 30% were sequences of influenza viruses isolated from the USA (Figure 2B). This large percentage would be associated with sampling bias due to the high surveillance activity in the United States [16]. Moreover, from Figure 2C, it can be seen that there is a large gap between the numbers of the nucleotide sequences isolated before 1991 and after 1992. This is not because the number of infections drastically increased after 1992 but because the use of the PCR technique became widespread around 1992. Thus the number of sequences is not associated with the number of infections by these viruses.

**Figure 3:**
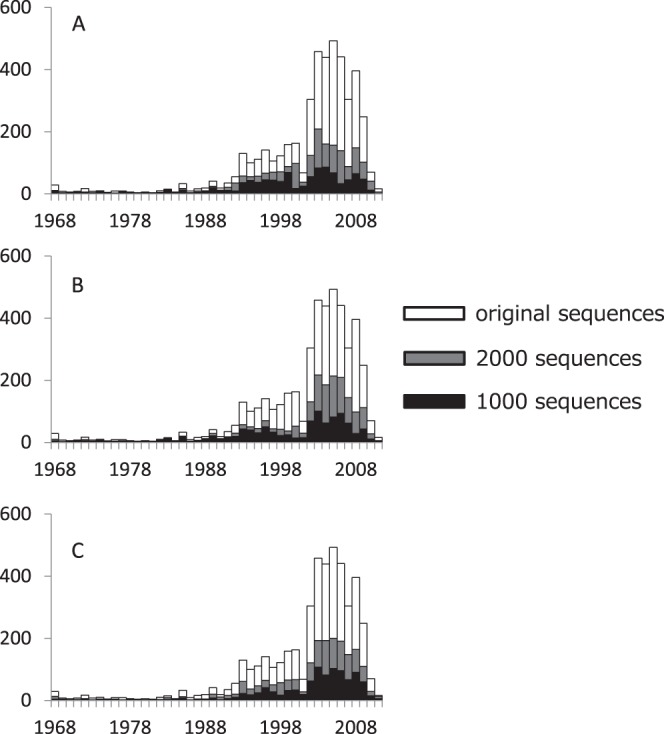
Changes of the distribution of the isolation years by means of (A) CNT, (B) NHC, and (C) kMC.

Figure 3 shows the changes of distributions of isolation years as CNT, NHC, and kMC proceed with trimming. This figure demonstrates that all of them flatten the distributions of isolation years. In the following section we will present more precise analyses.

### Evaluation of Resampled Sequences

In the following analyses, we executed the algorithms with randomization, namely kMC, 100 times each and calculated the average maximum similarities, nucleotide diversities, and standard deviations for the number of resampled sequences in a year.

#### Average maximum similarities of discarded sequences to the resampled sequences

**Figure 4:**
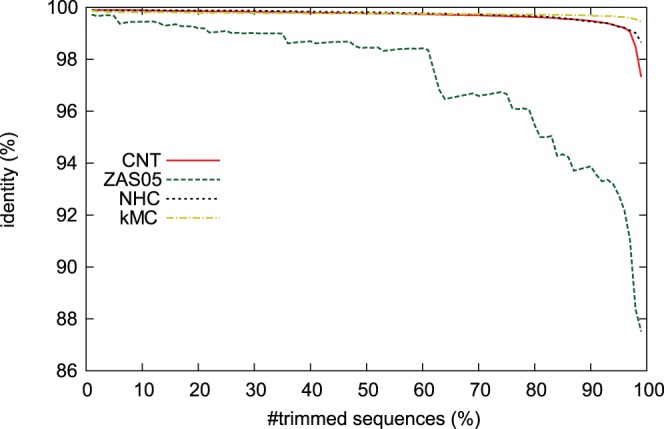
Relationship between average sequence identity between 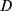 and 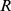 sequences and the fraction of discarded sequences. The horizontal axis represents the percentage of discarded sequences and the vertical axis represents identity 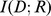. It can be seen that the performance of ZAS05 is worse than those of the other three algorithms.

First we investigated the average sequence identity of discarded sequences against resampled sequences. Let 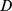 and 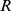 be the sets of the discarded and the remaining sequences, respectively. If a resampling algorithm discards one of the densely sampled sequences, 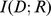, the average maximum identity from the discarded sequences to the remaining ones, is expected to remain closer to 100%. Since the lowest identity among all pairs of nucleotide sequences was larger than 82.3%, no pair of sequences had an identity lower than 82.3%. Thus the average of maximum identity of the discarded sequences to the resampled sequences, 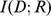, never becomes smaller than 82.3%. Figure 4 shows the values of 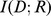 against the numbers of the discarded sequences. 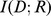 of the CNT, NHC, and kMC algorithms remained near 100% until 90% of the sequences were discarded. On the other hand, 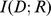 of ZAS05 fell more quickly than with the other three resampling algorithms. Therefore, in the following analysis, we excluded ZAS05.

#### Nucleotide diversities of resampled sequences

**Figure 5:**
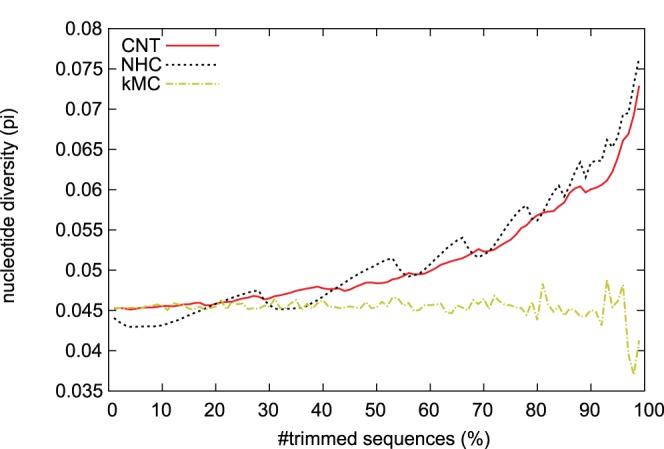
Relationship between nucleotide diversity 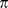 and the fraction of discarded sequences.

Figure 5 shows the relationship between 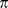 and the fraction of discarded sequences. As trimming proceeded, the diversity of the sequences resampled by CNT or NHC increased. On the other hand, the diversity of the sequences resampled by kMC remained similar to the original value. We consider that the reason for this discrepancy is that the kMC algorithm does not always select medoids from densely sampled sequences, and this would be a disadvantage of the kMC algorithm. The NHC algorithm shows good performance with respect to 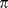 but it fluctuates. A possible reason is the fact that there are many possible pairs of sequences that have the same Hamming distances. The diversity 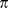 increases or decreases, depending on the sequence diversity around the removed sequences. When NHC is processing a pair in densely sampled clusters, the sequence diversity increases. But when it processes a pair in sparsely sampled ones, the sequence diversity decreases even if the pair has the smallest Hamming distance. Our implementation of NHC processes the first pair found in the dataset when there is more than one pair of sequences that have same Hamming distance. This is the cause of the fluctuation seen in the result for the NHC algorithm.

#### Standard deviations for the number of resampled sequences in a year

**Figure 6:**
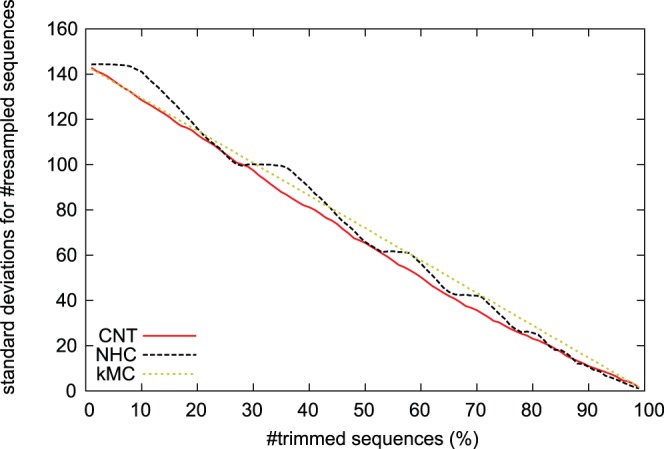
Standard deviations for the number of resampled sequences in a year. In each figures, the horizontal axis represents the ratio of discarded sequences. The vertical axis represents standard deviation for the number of resampled sequences in a year.

We focus on the statistics of resampled sequences from 1968 to 2011. In the original dataset, the average number of sequences for one year was about 106, with a standard deviation of around 142. As described in the background section, the database had more recent sequences. This large standard deviation is due to sampling bias, because most of the sequences in the dataset were derived from viruses isolated after 1992, as the dataset contained fewer sequences before 1991. The standard deviation of the number of sequences decreases almost linearly as more sequences are discarded by all of the resampling algorithms (Figure 6). For example, when the CNT trimmed 80% of the sequences, the average number of sequences for one year was about 21, with a standard deviation of around 23. This result indicated that the kMC algorithm had the worst performance in removing densely sampled sequences from the dataset.

### Evaluation of Execution Time

To evaluate the computational cost of the CNT algorithm, we measured the total execution times of the CNT, NHC, kMC, and ZAS05 algorithms (Table 1). The CNT and ZAS05 methods need to construct a phylogenetic tree from the given sequence dataset before resampling. The execution time was measured using a resampling task that selected 1000 of the 4655 sequences in the dataset. The phylogenetic trees constructed from the resulting 1000 sequences showed similar shapes and topologies (Figure S2). As can be seen in Table 1, the kMC algorithm was the fastest among the four algorithms. The CNT and ZAS05 algorithms take longer to process because they need to construct a phylogenetic tree before resampling.

**Table 1:** Execution time of the four resampling algorithms against the nucleotide sequences of human H3N2 influenza virus with 1000 sequences.

	algorithm			
	CNT	ZAS05	NHC	kMC
Constructing adistance matrix	183	183	183	183
Constructing a tree	1072	1072	0	0
Resampling	54	2011	198	1
Reconstructinga tree	16	16	16	16

The time units are seconds.

## Discussion

Due to the large amount of genetic information accumulated in public databases, researchers have to wait a long time, when conducting analyses using whole datasets. Compact subsets of nucleotide sequences can be obtained by resampling algorithms, and the subsets could reduce the computational time needed for the analyses. Sampling bias may affect the results of computational analyses using a large number of nucleotide sequences. If we can remove the sampling bias contained in datasets, more correct analyses could be achieved than those using the original datasets. Thus we believe that the capability for reducing sampling bias is more important than execution time for resampling algorithms.

In this paper, we proposed a novel resampling algorithm–called the closest-neighbor trimming (CNT) method–that removes densely sampled sequences from a given dataset. We discussed the performance of our algorithm, comparing it with three other algorithms. With respect to the average maximum similarities of discarded sequences to the resampled sequences (see Figure 4), ZAS05 seems less useful for resampling from a large number of nucleotide sequences than CNT, NHC, and kMC. From the experiment on the nucleotide diversity 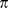 (see Figure 5), kMC looks less powerful than CNT and NHC. Measuring standard deviations for the number of resampled sequences in a year shows that CNT is more useful for reduction of sampling bias than NHC. In fact, Figure 3A shows that the nucleotide sequences resampled by CNT have more balanced distribution than the original ones. Therefore, we conclude that the CNT algorithm can be used for resampling nucleotide sequences in large datasets.

The dataset we used consisted of 4655 sequences of 984 nucleotides. Our method is applicable to any kind of nucleotide sequence dataset as long as the dataset can produce a reasonable phylogenetic tree.

We consider that the main reason for its superior performance is that the CNT algorithm tends to remove densely sampled new sequences and to conserve sparsely sampled old sequences. Because of the sparseness of sequences, there are more short pairs of neighbors of densely sampled new sequences than of sparsely sampled old sequences. Therefore the CNT method tends to trim newer sequences in the early steps.

It is difficult to select which of the closest neighbors to be trimmed with the CNT algorithm. In the case that no outlier sequence is included in the dataset, CNT should trim the closest neighbor with the shorter length to the parent. Moreover, CNT-further preserves the overall length of the phylogenetic tree whereas CNT-closer may shrink the tree. However, CNT-further resamples outlier sequences. This might harm the performance of the CNT-further.

One may wonder how many sequences should be discarded when analyzing a dataset. However, we have no clear answer for this question because the proper number of sequences to be discarded depends on what the user wants to do in the subsequent analyses. It might be proper to set the threshold where the nucleotide diversity 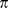 of the trimmed data is the highest.

**Figure 7:**
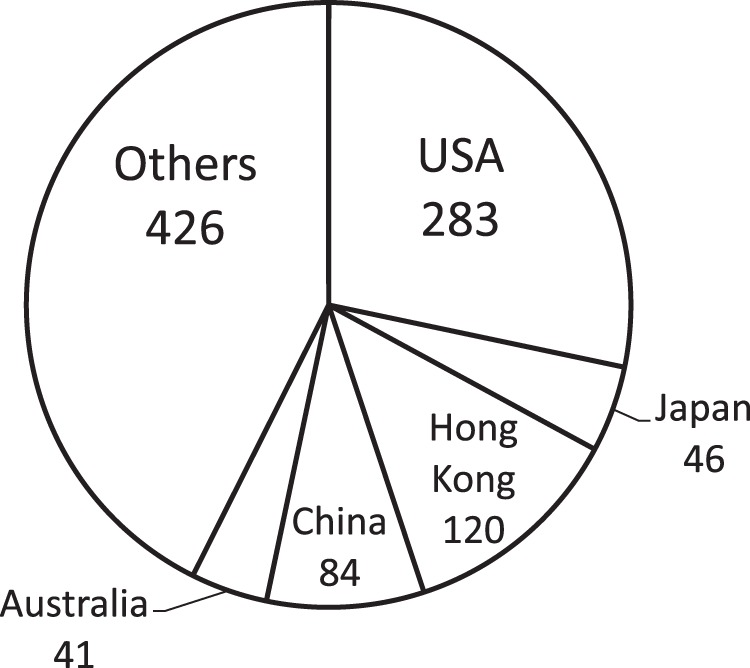
Distribution of the isolation countries for 1000 sequences resampled by the CNT algorithm.

In this paper, a dataset consisting of nucleotide sequences of human H3N2 influenza viruses was used to evaluate resampling algorithms. Through the resampling tests, we found an interesting phenomenon. When we resampled 1000 of 4655 sequences with CNT, the ratios of sequences of influenza viruses isolated from Hong Kong and China increased and those of viruses from the USA and Japan decreased (Figure 7). These results lead to two hypotheses. One is that USA and Japan had higher surveillance activities than other countries and that the sequences from these two countries were sampled more densely than for other countries. The other is that China has a large variation of influenza A viruses.

### Conclusion

In this paper, we proposed a novel algorithm. The proposed method, called the closest-neighbor trimming method, thins out nucleotide sequences by trimming a phylogenetic tree. The performance of our algorithm was compared with other algorithms by using the nucleotide sequences of human H3N2 influenza viruses. We have demonstrated that the CNT algorithm can be used to remove densely sampled sequences from a given dataset, together with removing sampling bias. Since nucleotide sequences are among the most widely used material for life science, the application of our algorithm to various datasets is expected to be useful for reducing sampling bias.

## Supporting Information

Figure S1
**Performances of the resampling results including the CNT-shorter and the CNT-random methods, (A) identities 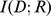, (B) nucleotide diversities 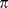, and (C) standard deviations for the number of resampled sequences in a year.** In (C), the median values are indicated by the center lines. The top and bottom edges of each box mark indicates the first and the third quatile, respectively. The whiskers extending from the box indicate the highest and lowest values.(PPT)

Figure S2 **Phylogenetic trees with (A) the original dataset with 4655 sequences and the resampling results of (B) CNT, (C) ZAS05, (D) NHC, and (E) kMC, with 1000 sequences.** All trees were drawn using Dendroscope [17].
(PPT)
