# Supplementary figures and images for: Resampling Nucleotide Sequences with Closest-Neighbor Trimming and Its Comparison to Other Methods
Source: PLoS One. 2013 Feb 27;8(2):e57684. doi: 10.1371/journal.pone.0057684 (PMC3583903; doi:10.1371/journal.pone.0057684)

## Slide 1
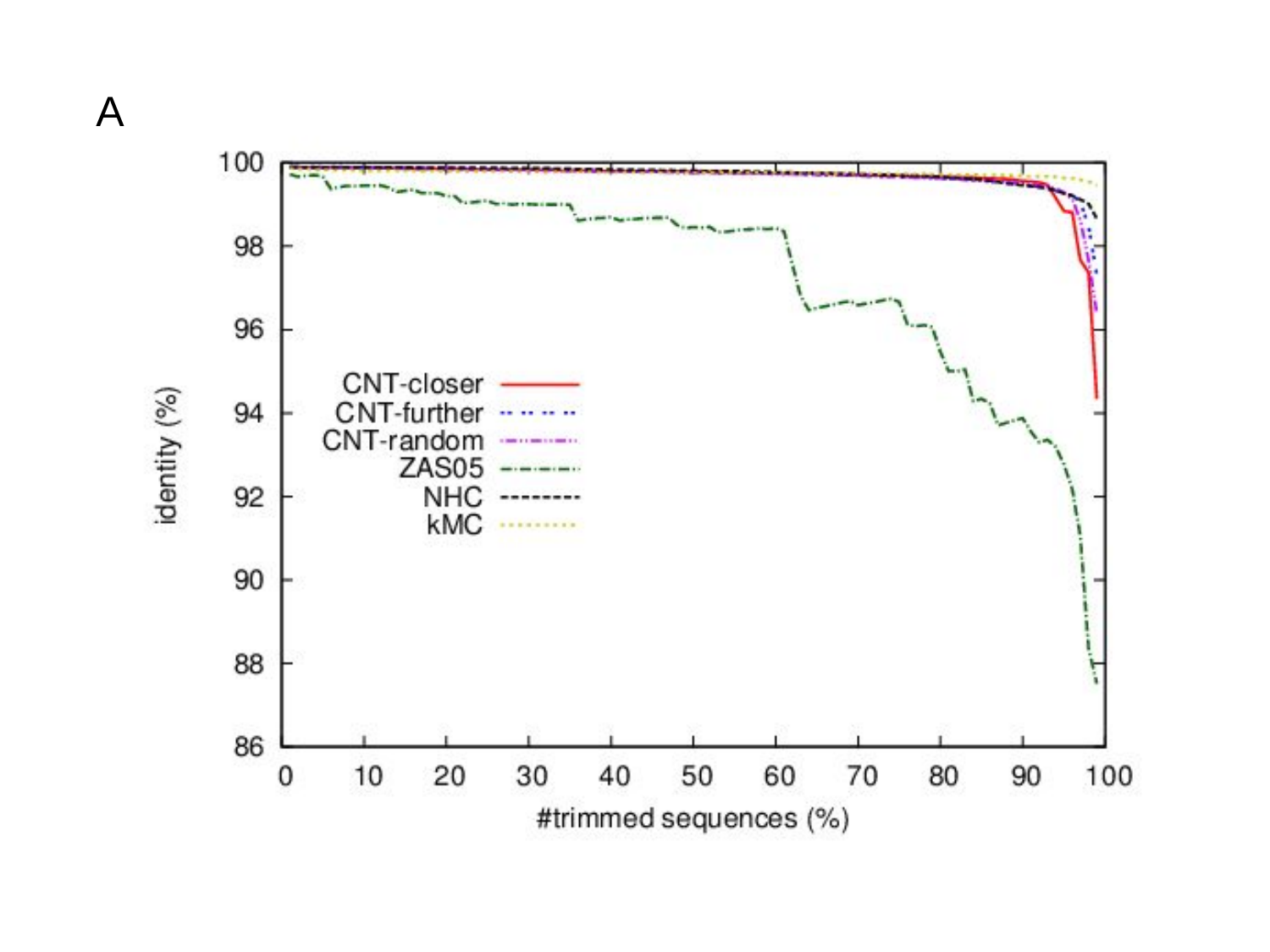

A

## Slide 2
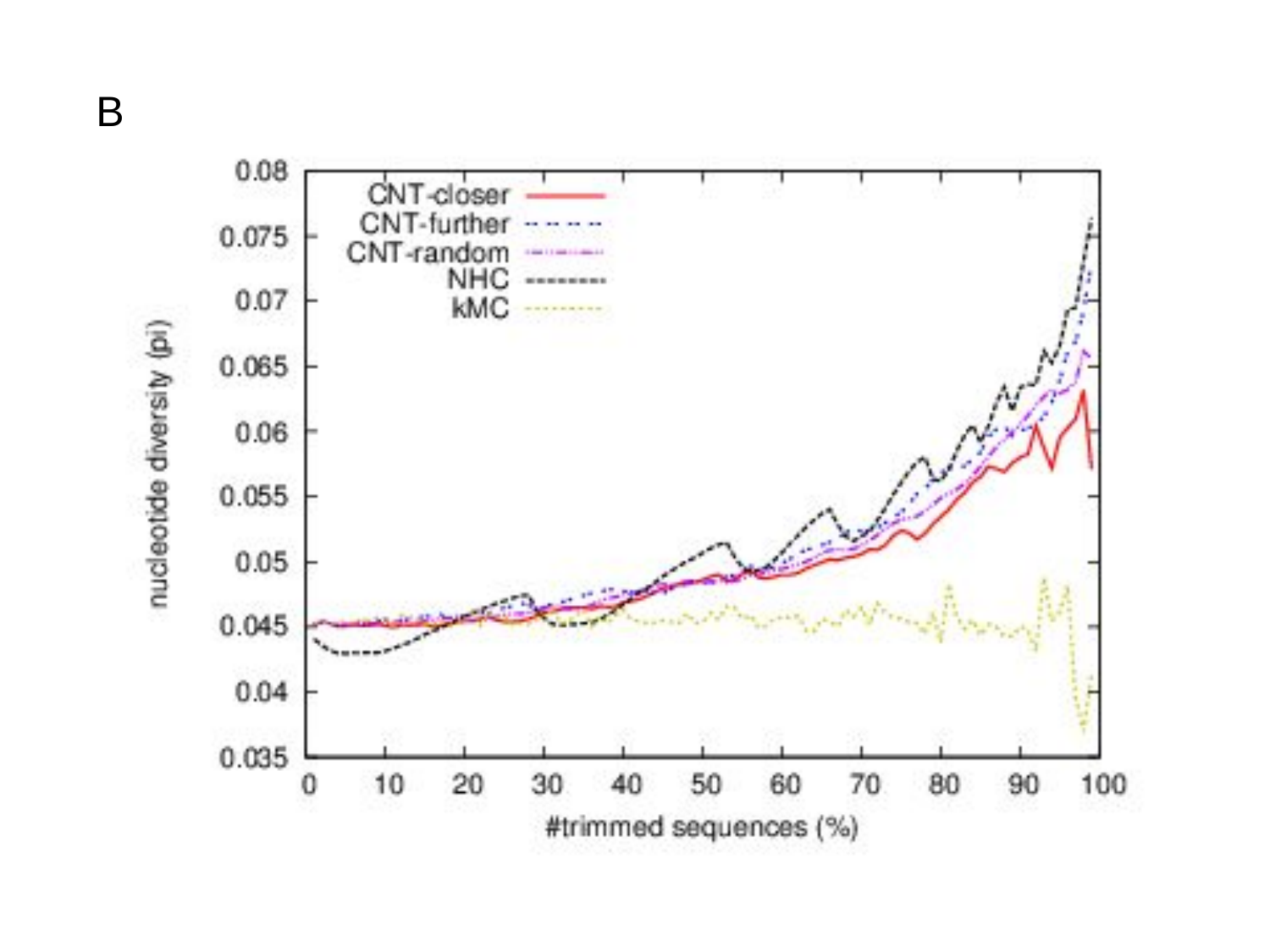

B

## Slide 3
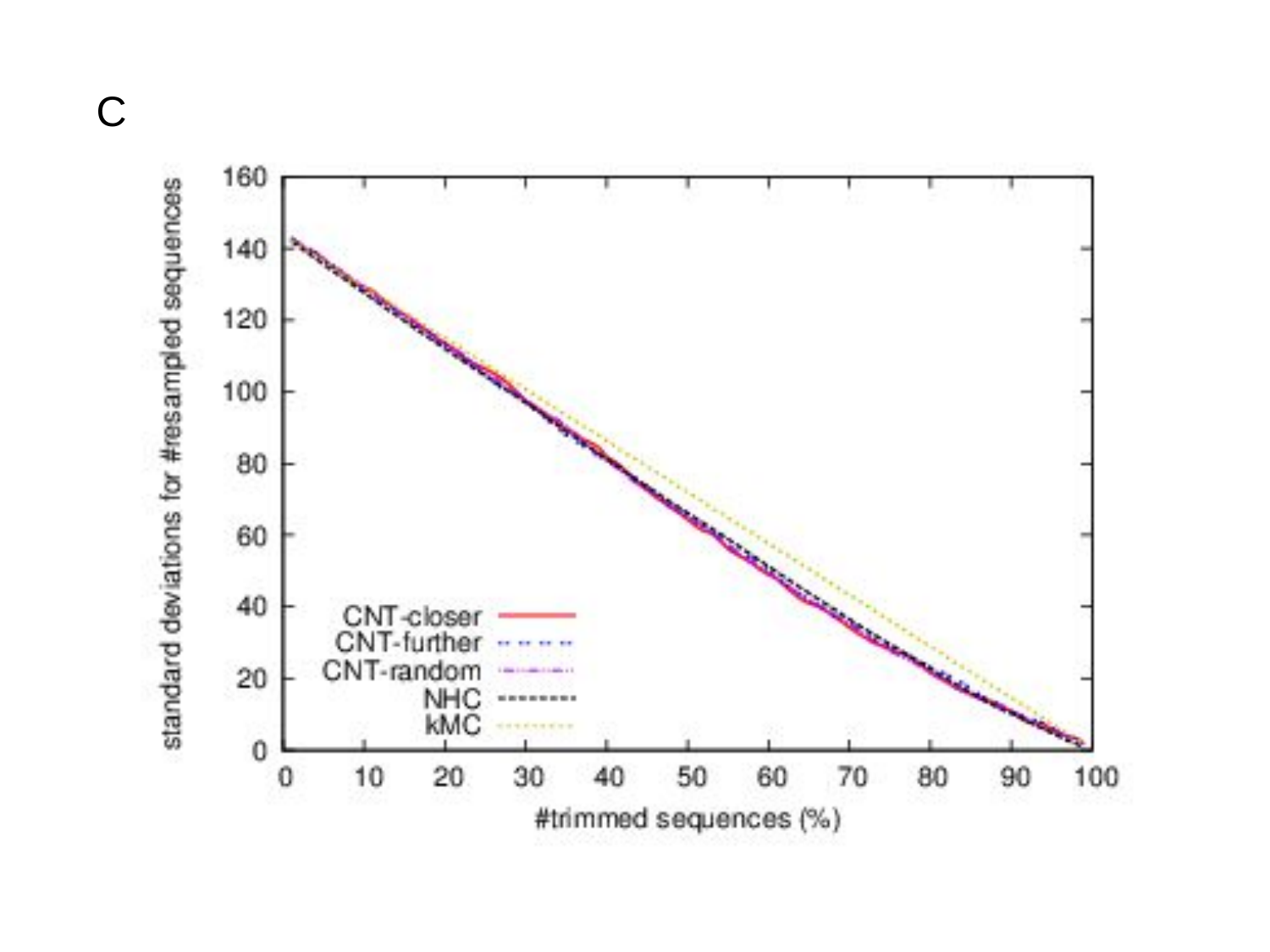

C

Supplement: Figure S1 — Performances of the resampling results including the CNT-shorter and the CNT-random methods, (A) identities , (B) nucleotide diversities , and (C) standard deviations for the number of resampled sequences in a year. In (C), the median values are indicated by the center lines. The top and bottom edges of each box mark indicates the first and the third quatile, respectively. The whiskers extending from the box indicate the highest and lowest values. (PPT) [file pone.0057684.s001.ppt]

## Slide 1
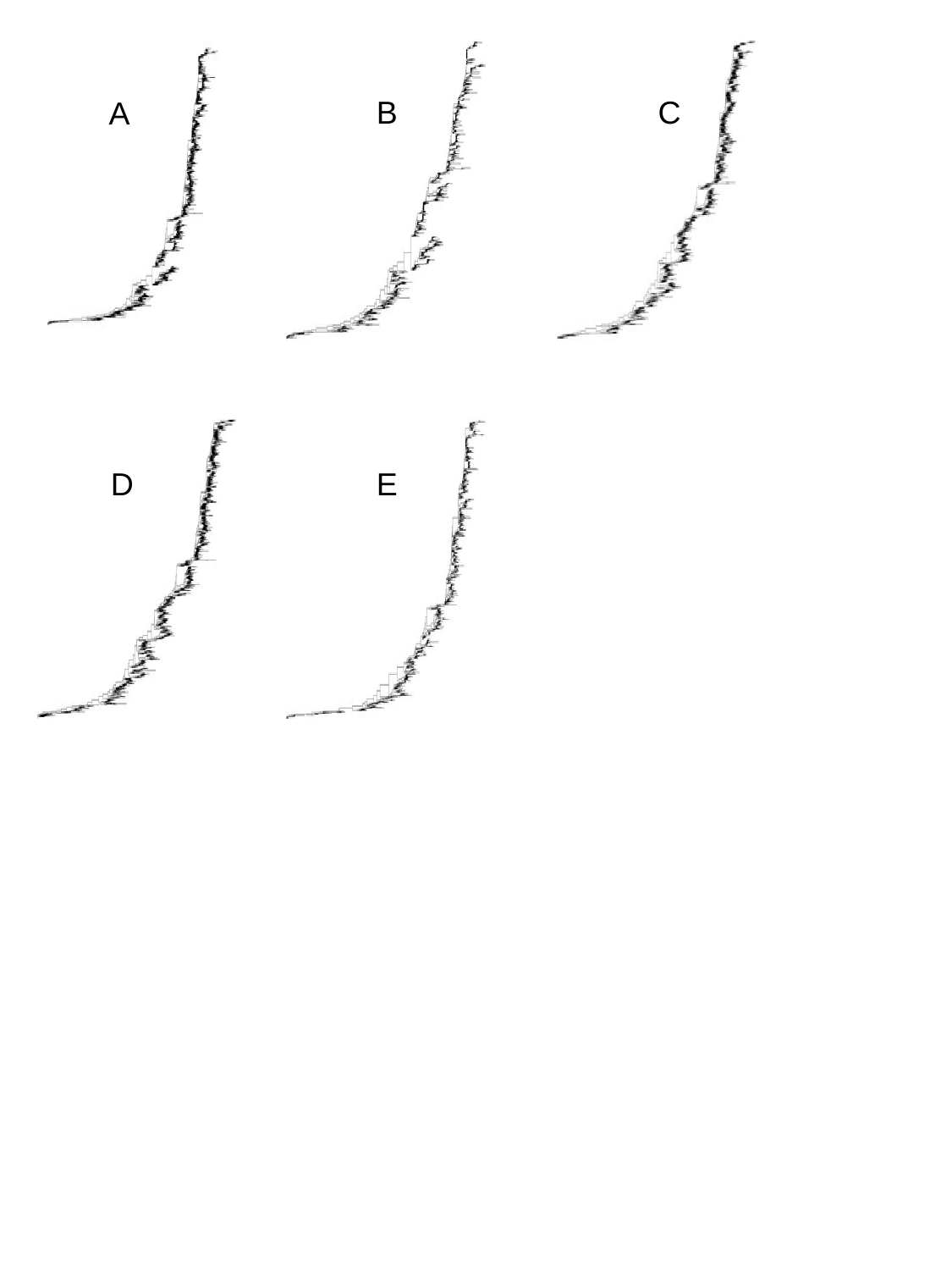

B
C
A
D
E

Supplement: Figure S2 — Phylogenetic trees with (A) the original dataset with 4655 sequences and the resampling results of (B) CNT, (C) ZAS05, (D) NHC, and (E) kMC, with 1000 sequences. All trees were drawn using Dendroscope [17]. (PPT) [file pone.0057684.s002.ppt]
